# Pertussis outbreak in neonates and young infants across Italy, January to May 2024: implications for vaccination strategies

**DOI:** 10.2807/1560-7917.ES.2024.29.23.2400301

**Published:** 2024-06-06

**Authors:** Marco Poeta, Cristina Moracas, Chiara Albano, Laura Petrarca, Marco Maglione, Luca Pierri, Maurizio Carta, Paolo Montaldo, Elisabetta Venturini, Maia De Luca, Danilo Buonsenso, Ilaria Brambilla, Vania Giacomet, Andrea Lo Vecchio, Eugenia Bruzzese, Fabio Midulla, Claudia Colomba, Alfredo Guarino

**Affiliations:** 1 Department of Translational Medical Science, University of Naples “Federico II”, Naples, Italy; 2 Pediatric Infectious Disease Unit, Department of Maternal and Child health, University Hospital “Federico II”, Naples, Italy; 3 PhD National Program in One Health approaches to infectious diseases and life science research, Department of Public Health, Experimental and Forensic Medicine, University of Pavia, Pavia, Italy; 4 Department of Health Promotion, Maternal and Infant Care, Internal Medicine and Medical Specialties, University of Palermo, Palermo, Italy; 5 Department of Maternal, Infantile and Urological Sciences, Sapienza University of Rome, Rome, Italy; 6 Pediatric Emergency Department, Santobono-Pausilipon Children’s Hospital, Naples, Italy; 7 Neonatal Intensive Care Unit and Neonatology, Emergency Department, Santobono-Pausilipon Children’s Hospital, Naples, Italy; 8 Neonatology and Neonatal Intensive Care Unit, University Hospital Policlinico “Paolo Giaccone”, Palermo, Italy; 9 Department of Woman, Child, and General and Specialized Surgery, University of Campania “Luigi Vanvitelli,” Naples, Italy; 10 Infectious Diseases Unit, Meyer Children’s Hospital, IRCCS, Florence, Italy; 11 Infectious Disease Unit, Bambino Gesù Children’s Hospital, IRCCS, Rome, Italy; 12 Pediatric Infectious Diseases and Clinical Pediatric Ultrasound, Department of Woman and Child Health and Public Health, Fondazione Policlinico Universitario A. Gemelli IRCCS, Rome, Italy; 13 Department of Clinical, Surgical, Diagnostic and Pediatric Sciences, Fondazione IRCCS Policlinico San Matteo, Pavia, Italy; 14 Department of Pediatrics, Pediatric Infectious Disease Unit, L. Sacco Hospital, University of Milan, Milan, Italy; 15 Division of Pediatric Infectious Diseases, “G. Di Cristina” Hospital, ARNAS Civico Di Cristina Benfratelli, Palermo, Italy

**Keywords:** Whooping cough, *Bordetella pertussis*, outbreak, epidemic, newborns, infants, vaccination, maternal immunization, post-exposure prophylaxis

## Abstract

Since January 2024, Italy experiences a pertussis outbreak, primarily affecting neonates and unvaccinated infants at high risk of severe complications and mortality; 11 major paediatric centres noted 108 hospitalisations and three deaths by 10 May. The outbreak reflects increased circulation of *Bordetella pertussis* and non-adherence to immunisation recommendations during pregnancy. Public health interventions, including maternal immunisation, vaccination of infants as early as possible and post-exposure prophylaxis, are critical for reducing the burden of pertussis and preventing further mortality.

Following the COVID-19 pandemic, the resurgence of pertussis (whooping cough) in Europe since the end of 2023 represents a pressing public health concern. This phenomenon has been observed in the European Union/European Economic Area (EU/EEA) and the United Kingdom (UK), with some countries reporting pertussis-related deaths [1]. Here, we present data on an ongoing pertussis outbreak in Italy, from January up to May 2024, affecting neonates and young infants.

## Data sources

All presented data were collected through a paediatric surveillance system established within the network of an EU-funded National Recovery and Resilience Plan (NRRP) project, the One Health Basic and Translational Actions Addressing Unmet Needs on Emerging Infectious Diseases (INF-ACT). This initiative aims to identify infectious threats and investigate emerging and re-emerging infections in the post-COVID-19 era through monthly online meetings involving clinicians from seven Italian reference centres. To monitor epidemic trends, one clinician from each of the seven Italian reference centres and from four additional paediatric/neonatal wards across Italy not included in the INF-ACT network filled out an online form (survey) detailing the number of admitted pertussis cases aged 0–24 months, the vaccination status, the number receiving oxygen or ventilation, intensive care unit (ICU) admissions and any recorded deaths. In addition to the survey, detailed information of hospitalised children was available from three centres located in Naples, Rome and Palermo (referred to as the ‘study cohort’).

## Survey results of pertussis cases

**Figure 1:**
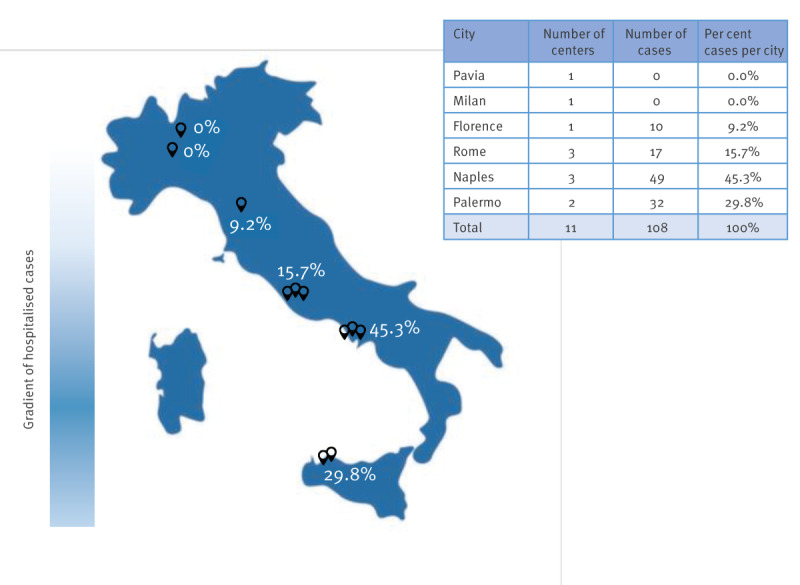
Distribution of hospitalised pertussis cases aged 0–24 months at 11 participating centres, Italy, 1 January–10 May 2024 (n = 108)

**Figure 2:**
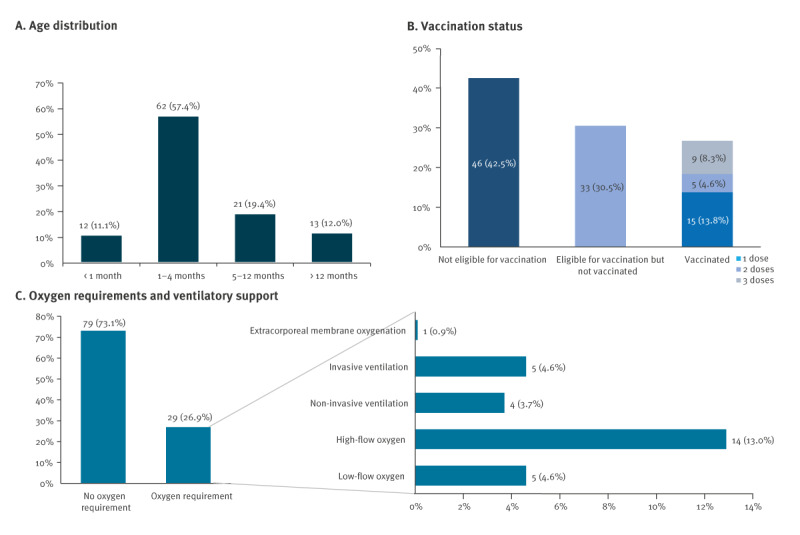
Characteristics of hospitalised pertussis cases aged 0–24 months, Italy, 1 January–10 May 2024 (n = 108)

A total of 108 children with pertussis were hospitalised at the 11 participating centres from 1 January 2024 to 10 May 2024. Compared with 2022 (n = 5) and 2023 (n = 12), we observed an 800% increase in the number of hospitalised cases within just 4 months of the start of 2024, with a higher number of cases in Naples (n = 49; 45.3%) and Palermo (n = 32; 29.8%), both cities in the south of Italy (Figure 1). All children had a respiratory sample positive for *Bordetella pertussis* identified by RT-PCR. No cases tested positive for *B. parapertussis*. Median age was 3 months (interquartile range (IQR): 1.5–4.8). Most were neonates (0–1 month) or young infants 1–4 months of age (n = 74; 68.5%) (Figure 2A). As shown in Figure 2B, most were unvaccinated for pertussis.

The shading of the left bar indicates an increasing gradient of hospitalised cases from the north to the south of Italy.

Case numbers and per cent of total are provided at the top/end of each bar.

Types of ventilatory support are outlined in Figure 2C. Twelve infants (11.1%) required ICU admission (median age: 2 months, range: 0.5–3). Two children died after complications arising from pertussis: one neonate, aged 25 days, developed pulmonary hypertension necessitating extracorporeal membrane oxygenation (ECMO) and presented coinfection with rhino/enterovirus; the other, aged 30 days, died following a leukaemoid reaction previously treated by exsanguino-transfusion. A third death, involving a 15-day-old neonate, was notified through national daily reporting during the same period by another centre not included in the INF-ACT network.

## Study cohort characteristics

Detailed information on demographics, clinical, biochemical parameters, imaging and treatments was collected for a hospitalised cohort of 75 children (median age: 3 months, IQR: 2–4.2) from three centres located in Naples, Rome and Palermo (Table).

**Table:** Demographic, clinical, laboratory and radiological features and main treatments of the study cohort, Italy, 1 January–10 May 2024 (n = 75)

Characteristics	Study population (n = 75)	
	n	%
Demographic features		
Male sex	43	57.3
Female sex	32	42.7
Age at admission in months, median (IQR)	3.0 (2–4.2)	
Risk and protective factors		
Mother vaccinated during pregnancy	3/53	5.7
Mother informed about pertussis vaccination	10/48	20.8
At least one pertussis vaccination	14	18.7
Household contacts with respiratory symptoms	15/50	30.0
At least one coinfection	47	62.7
Previous visit to a healthcare setting	20/42	47.6
Clinical features		
Fever	13	17.3
Cough	75	100
Number of cough paroxysms/day, mean (SD)	13.6 (6.7)	
Cyanosis	45	60.0
Rhinorrhoea	18	24.0
Respiratory distress	20	26.7
Vomiting	13	17.3
Length of hospital stay, mean days (SD)	6.5 (6.1)	
Biochemical parameters, mean (SD)^a^		
Haemoglobin, g/dL	11.9 (1.4)	
WBC, cells/µL	20,728 (13,684)	
Neutrophils, cells/µL	5,711 (5,263)	
Lymphocytes, cells/µL	13,695 (9,824)	
Platelet count, x10^3^ cells/µL	537.2 (176)	
CRP, mg/L	4.4 (10.6)	
Procalcitonin, ng/mL	0.1 (0.3)	
Chest X-ray		
Abnormal findings	11/22	50.0
- Lobar	7/22	31.8
- Interstitial	4/22	18.2
Treatment		
Parenteral rehydration	29	38.7
Oxygen supplementation	21	28.0
Clarithromycin^b^	66	88.0
Azithromycin^c^	6	8.0
Trimethoprim-sulfamethoxazole^d^	2	2.7
Severity [2]		
PSS > 5	34	45.3

CRP: C-reactive protein; PSS: pertussis severity score; SD: standard deviation; WBC: white blood cells.

^a^ Reference values for biochemical parameters (children < 24 months) are haemoglobin: 10.5–13.5 g/dL; WBC: 6.0–17.0 cells/µL; neutrophils: 1.5–8.5 cells/µL; lymphocytes: 4.0–10.5 cells/µL; platelet count: 150–450 cells/µL; CRP: < 5 mg/L; procalcitonin: < 0.5 ng/mL.

^b^ Dose: 15 mg/kg/day in two oral doses for 7 days.

^c^ Dose: 10 mg/kg/day as a single oral dose for 5 days.

^d^ Dose: 8 + 40 mg/kg/day in two oral doses for 14 days.

Denominators are provided where data are available for only a subset of the 75 cases in the study cohort.

A household contact with respiratory symptoms was reported in 15 of 50 cases with available information, and 20 of 42 had visited a healthcare setting (i.e. paediatrician’s office, emergency room, previous hospitalisation) within 21 days before the onset of pertussis symptoms. Only 3 of 53 mothers for whom information was available had received pertussis vaccination during pregnancy, and only 10 of 48 had been informed about the possibility of vaccination during pregnancy (Table).

Paroxysmal cough was present in all 75 cases, cyanosis in 60% (n = 45), and respiratory distress in 26.7% (n = 20). Fever was uncommon (17.3%; n = 13). Children displayed a typical increase in lymphocyte count. Chest radiography was performed in 22 children, of which 11 showed abnormal findings. Clarithromycin was the most common antibiotic therapy (88.8%), and in two cases a second-line therapy was started because of clinical deterioration.

**Figure 3:**
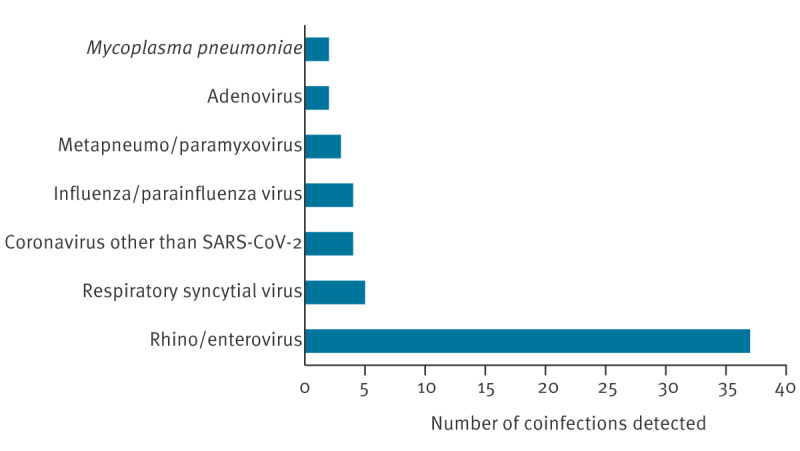
Distribution of coinfections in the study cohort, Italy, 1 January–10 May 2024 (n = 75)

Of the 75 pertussis cases, 47 (62.7%) had a coinfection, predominantly with a virus (Figure 3). No significant differences were found between cases with mono-infection and those with coinfections, except for a tendency to higher oxygen requirements in children infected with a second pathogen (15/47 vs 6/28).

SARS-CoV-2: severe acute respiratory syndrome coronavirus 2.

Thirty-four patients (45.3%) experienced severe infection, defined by a pertussis severity score (PSS) > 5 [2] (Table). They exhibited a comparable frequency of coinfections (55.9% vs 68.3%; chi-square p = 0.193), but a higher lymphocyte count (16,547 ± 11,558 vs 11,342 ± 7,481; t-test p < 0.05) and C-reactive protein (CRP) values (7.5 ± 13.6 vs 2.0 ± 6.4 mg/L; t-test p < 0.05).

Infants under 4 months of age presented a longer hospital stay (7.3 ± 7.1 vs 5.1 ± 3.5 days; t-test p < 0.05) and duration of fever (2.2 ± 0.8 vs 1.4 ± 0.5 days; t-test p < 0.05), and a higher number of paroxysms per day (15.1 ± 7.5 vs 11 ± 3.9; t-test p < 0.05).

Compared with the study cohort, the three children born to mothers vaccinated during pregnancy exhibited a shorter length of hospital stay (3.7 ± 0.6 days), and none required oxygen supplementation.

## Discussion

Since the end of 2023/beginning of 2024, Italy has witnessed a notable increase in hospitalised paediatric pertussis cases. This marks the first surge of pertussis in the post-COVID-19 era and represents the largest whooping cough outbreak in recent decades. The COVID-19 pandemic restrictions, particularly masking and physical distancing measures, disrupted the usual patterns of distribution of common respiratory pathogens such as influenza and RSV [3], and negatively impacted vaccine coverage in many countries. Similarly, COVID-19 mitigation strategies led to a significant decrease in *B. pertussis* circulation, potentially compromising population immunity [4].

In Italy, the primary immunisation cycle against pertussis consists of three doses given at 3, 5 and 11 months of age, and followed by booster doses in preschool age (at 6 years), in adolescents (12–18 years), and in adults, to be repeated every 10 years. Unlike other countries, the Italian outbreak primarily affects a high proportion of newborns and young infants who are either unvaccinated or incompletely vaccinated. In contrast, the epidemic in Denmark showed the highest incidence among adolescents [5]. This is probably due to different rates of vaccine coverage during pregnancy; while ca 85% of pregnant women in Denmark are vaccinated, vaccination coverage in the Italian population of pregnant women is unknown because of the absence of a national registry for prenatal vaccinations.

Moreover, a remarkable topographical concentration in the number of hospitalised cases was found across the country, with the highest proportions in southern Italy, probably reflecting differences in vaccination rates, although confirmed data for maternal vaccination coverage are lacking. However, as many as 94% of the mothers were unvaccinated, and 80% of them did not receive any information during pregnancy about prenatal vaccination, which underscores the need to implement targeted campaigns. Nevertheless, the lack of data on the vaccination status and awareness regarding maternal vaccination among the general population of pregnant women is a limitation of the study. Future research efforts aimed at addressing this gap are warranted to offer a more comprehensive understanding of the need and the effectiveness of targeted campaigns. Indeed, the priority is to protect infants too young to receive vaccinations, who are at the highest risk of severe complications [6]. During the current outbreak, three pertussis-related deaths were reported. All fatalities involved newborns who underwent a severe course of infection and were therefore too young to start the pertussis vaccine schedule [7]. Case-fatality rates are as high as 1.6% in infants < 2 months and 1.2% in infants 2–11 months of age [8]. In addition, nearly all mortality occurs in infants < 3 months, accounting for 90–100% of total deaths [9]. For this reason, the Global Pertussis Initiative endorsed the administering of the pertussis vaccine during the third trimester of pregnancy to mitigate this risk, preventing infection, hospitalisation and mortality in unvaccinated infants [10-12].

Within our study cohort, three infants born to vaccinated mothers exhibited a milder disease course, as evidenced by low severity score, no oxygen requirement and shorter hospital stays [13]. A Spanish ecological study examining changes in disease severity following the introduction of prenatal vaccination, identified the most significant beneficial effects in infants aged 0–2 months, who experienced an annual reduction in hospitalisation rates of 34% and a decrease in the length of hospital stay by 4 days [14].

Although no differences were observed between patients with mono-infection and those with coinfections, two out of the three children who died were infected with a second pathogen, suggesting the potential role of coinfection in disease severity, as previously reported [2,15].

In our study, 30% of children had symptomatic household contacts. Vaccination or infection does not confer lifelong immunity, leaving adolescents and adults susceptible to infection, and potentially becoming sources of transmission to vulnerable populations.

Furthermore, almost 50% of children had accessed a healthcare setting before the onset of disease, suggesting that potentially one of two pertussis cases could have been detected earlier, thereby preventing potential secondary cases. Therefore, it is essential to identify and isolate cases in healthcare facilities to limit transmission among pairs and recommend post-exposure prophylaxis (PEP) for those at high risk of severe course or in close contact with them [16,17].

## Conclusion

To effectively counteract the resurgence of pertussis, several strategies should be considered. These include implementing prenatal vaccination of mothers, vaccinating infants as early as possible, providing antibiotic PEP for contacts and starting vaccination campaigns targeting people who have missed booster doses. Additionally, active screening for individuals with respiratory symptoms should be promoted thus minimising exposure and containing the spread of the infection. The high number of hospitalised infants and reported mortality underscore the urgent need to address this public health concern.
